# Long Non-Coding RNAs, Nuclear Receptors and Their Cross-Talks in Cancer—Implications and Perspectives

**DOI:** 10.3390/cancers16162920

**Published:** 2024-08-22

**Authors:** Prabha Tiwari, Lokesh P. Tripathi

**Affiliations:** 1 Department of Microbiology and Immunology, Keio University School of Medicine, Shinjuku, Tokyo 160-8582, Japan; 2 Laboratory for Transcriptome Technology, RIKEN Center for Integrative Medical Sciences, Yokohama 230-0045, Kanagawa, Japan; 3 AI Center for Health and Biomedical Research (ArCHER), National Institutes of Biomedical Innovation, Health and Nutrition, Kento Innovation Park NK Building, 3-17 Senrioka Shinmachi, Settsu 566-0002, Osaka, Japan

**Keywords:** gene regulation, non-coding RNA, lncRNA, nuclear receptors, biological cross-talks, cancer signaling, cancer, gene regulatory networks

## Abstract

**Simple Summary:**

Long non-coding RNAs (lncRNAs) exert their influence on transcriptional regulation and genome organization by associating with transcription factors, chromatin, RNAs and various proteins. In particular, studies have established a widespread involvement of cross-talks between lncRNAs and nuclear receptors (NRs) in pathology of diseases such as cancer. A deeper understanding of these cross-talks will not only help gain greater insights into cancer physiology, but also guide the development for newer and improved therapeutic strategies aimed at cancer.

**Abstract:**

Long non-coding RNAs (lncRNAs) play key roles in various epigenetic and post-transcriptional events in the cell, thereby significantly influencing cellular processes including gene expression, development and diseases such as cancer. Nuclear receptors (NRs) are a family of ligand-regulated transcription factors that typically regulate transcription of genes involved in a broad spectrum of cellular processes, immune responses and in many diseases including cancer. Owing to their many overlapping roles as modulators of gene expression, the paths traversed by lncRNA and NR-mediated signaling often cross each other; these lncRNA-NR cross-talks are being increasingly recognized as important players in many cellular processes and diseases such as cancer. Here, we review the individual roles of lncRNAs and NRs, especially growth factor modulated receptors such as androgen receptors (ARs), in various types of cancers and how the cross-talks between lncRNAs and NRs are involved in cancer progression and metastasis. We discuss the challenges involved in characterizing lncRNA-NR associations and how to overcome them. Furthering our understanding of the mechanisms of lncRNA-NR associations is crucial to realizing their potential as prognostic features, diagnostic biomarkers and therapeutic targets in cancer biology.

## 1. Introduction

Non-coding RNAs (ncRNAs) are defined as transcripts with little or no protein-coding potential [1]. Genes encoding ncRNAs comprise a significant portion of the genome in higher eukaryotes. Until recently, ncRNAs were viewed as “junk” or “transcriptional noise”, but their biological functions have now been increasingly recognized. These functions include regulating gene expression, modulating the activities of transcription factors, chromatin remodeling, nuclear and cytoplasmic protein scaffolding and coregulation with other RNAs [1,2]. NcRNAs are broadly categorized as long non-coding RNAs (lncRNAs), which are typically defined as transcripts that are >200 nucleotides long [1], small ncRNAs such as microRNAs (miRNAs) and circular RNAs (circRNAs) [3]. Functions, protein binding properties and subcellular localization of lncRNAs typically correlate with short motifs (k-mers) rather than linear sequence homology [4]. Besides nuclear localization, lncRNAs can also associate with cytoplasm and cytoplasmic organelles such as mitochondria and ribosomes; they tend to acquire secondary and tertiary structures, which strongly correlate with their functions [5,6]. In general, lncRNAs can bind to DNA, RNA or proteins, are less conserved compared to other classes of RNAs [7,8], and their interactions may often be context-dependent or cell-type dependent [9]. These interactions enable lncRNAs to participate in a wide array of cellular processes in normal as well as in disease conditions [10,11,12]. In particular, lncRNA interactions with different transcription factors (TFs) play key roles in determining gene expression and are also implicated in pathophysiology of several human diseases [1,13,14]. Among the TFs, the nuclear receptors (NRs) frequently associate with lncRNAs; NRs are one of the largest groups of TFs that regulate the expression of functional genes involved in various biological processes such as development, immune responses and metabolism [15]. NRs are intracellular receptors; some NRs, including the androgen receptor (AR) and the glucocorticoid receptor (GR), are located in the cytoplasm and translocated to the nucleus after stimulation by agonists; other receptors such as retinoic acid receptors (RAR) are located in the nucleus and are bound to specific sequences even in the absence of ligands [16,17]. NRs are modulated by various endogenous ligands such as hormones, growth factors, fatty acids and their metabolites; although NRs with no known endogenous ligands, hence classified as orphan receptors, have also been reported [18]. Functional roles of NRs include transactivation or transrepression of target genes and coregulation of other transcriptional regulators [19]. NcRNAs can regulate NRs directly, or coregulate via direct interactions with NR-associated coactivators and corepressors, and also function as downstream targets of NRs in feedback loops [20,21]. Given the extensive and often overlapping involvement of lncRNAs and NRs in cellular physiology and diseases, investigating lncRNA-NR cross-talks is crucial to a deeper understanding of the pathogenesis of various diseases as well as in the identification of novel therapeutic targets [22]. In this review, we have specifically focused on how lncRNA-NR cross-talks drive cell proliferation, progression, tumorigenesis and metastasis in various types of cancer. We discuss the challenges in characterizing lncRNA-NR cross-talks in cancer systems and the probable approaches to generate more in-depth coverage of these associations. We finally deliberate on how a deeper understanding of lncRNA-NR cross-talks may assist in the development of newer and more effective diagnostics and anticancer therapies.

## 2. LncRNA Classification, Functional Characterization and Their Roles in Cancer

### 2.1. Functional Repertoire of lncRNAs

The complexity and diversity of lncRNA functions make their classification a challenging task [1]. Nevertheless, lncRNAs can be classified by genomic location (i.e., intergenic, intronic, sense, antisense), transcriptional regulation of nearby genes (i.e., cis, trans) and their effects on proteins (i.e., signal, decoy, guide, scaffold) [23]. LncRNAs can also be classified based on association with repeat elements or by their stability [24]. LncRNAs exert their functions primarily via biomolecular interactions, but also by undergoing structural modifications [1,25]. Therefore, a systematic characterization of lncRNA interactions with other biomolecules in the form of lncRNA interactome maps will help to better understand their biological functions [26]. Accordingly, genome-wide technologies have been developed to map system-wide RNA-chromatin interactions [27]. While secondary structure determination of lncRNAs is difficult due to variation in sizes and the heterogeneous nature of the transcripts, various methods such as enzymatic footprinting, chemical probing, nuclear magnetic resonance (NMR) spectroscopy and comparative sequence analysis have been employed to determine lncRNA secondary structure [6] and to correlate them with lncRNA function [28]. Functional roles played by lncRNAs include regulation of local gene expression in cis, at distant genomic locations in trans, epigenetic and enhancer regulation [10,29,30,31].

Regulation of gene expression by lncRNAs is a multilayered process and involves many different mechanisms. Broadly, lncRNAs modulate gene expression by (a) associating with chromatin and (b) by modulating RNA splicing, transcript stability and translation. Chromatin regulation by lncRNAs is facilitated by direct or indirect interactions (via proteins) between lncRNAs and specific-DNA regions, which facilitate the modification of the local chromatin environment and govern the changes in gene expression [32,33]. A key feature of direct interactions between lncRNAs and DNA is the formation of hybrid structures, namely RNA-DNA-DNA triplexes that are formed between RNAs and double-stranded DNA [34] and R-loops that are formed between RNA and single-stranded DNA [35]. Transcriptional and post-transcriptional regulation by lncRNAs variously involves gene silencing as in the case of XIST lncRNA [36], lncRNAs functioning as micro RNA (miRNA) sponges, thereby restricting the ability of miRNAs to target expressed mRNAs [37] or functioning as decoys of chromatin modifiers, and thus preventing them from associating with the target gene promoters [38]. A subset of lncRNAs, namely natural antisense transcripts that are transcribed from the opposite strands of the genes, are also deeply involved in gene regulatory processes [39]. Multiple studies have suggested key roles for lncRNAs in alternate splicing of genes that can have diverse implications for the development, differentiation and pathogenesis of various diseases such as cancer [40]. For a detailed discussion on the mechanisms of gene regulation by lnRNAs, readers are also encouraged to see the review on the subject from Statello and colleagues [41]. 

There is a widespread interest in the characterization of the lncRNA repertoire and their functions at the whole genome level. The FANTOM6 (Functional Annotation of Mammalian Genomes) initiative is aimed at a system-wide characterization of lncRNA functions in different cellular contexts and has provided novel insights into functional roles of many different lncRNAs [42,43]. There is also a growing cognizance of networks of extensive cross-talks between lncRNAs and other cellular factors, especially TFs such as NRs to determine gene expression, modulate signaling pathways and regulate cellular physiology [1,42,43].

### 2.2. LncRNAs in Cancer

There is an increasing acknowledgment of the involvement of lncRNAs in virtually all tumorigenesis and cancer progression-related pathways such as Wnt/β-catenin, Hippo, Notch, NF-κB and TGF-β that are involved in proliferation, differentiation, metastasis and apoptosis [44,45]. For instance, metastasis-associated lung adenocarcinoma transcript 1 (*MALAT1*) and nuclear-enriched abundant transcript 1 (NEAT1) are strongly implicated in cancer progression and metastasis in various cancer types [46,47,48]. *MALAT1* is overexpressed and displays increased mutations in many cancer types [49,50,51]. Moreover, various cell-cycle regulatory proteins involved in cancer progression such as cyclins/cyclin-dependent kinases and transcription factors (E2F, p53) are known to be regulated by lncRNAs [52]. Many cellular processes in cancer stem cells (CSCs) that are known to initiate tumor formation can be regulated by lncRNAs such as *MALAT1*, *ROR*, *HOTAIR*, *H19*, *UCA1* and *ARSR* [53,54]. Autophagy, which can play dual roles in both the promotion and inhibition of tumorigenesis, can be regulated by lncRNAs such as *NBR2*, *MEG3*, *PTENP1*, *GAS5*, *HULC* and *HOTAIR* [55,56]. Indeed, several autophagy-related lncRNAs having prognostic value were identified in bladder cancer [57]. Tumor microenvironment and peripheral immune landscape perturbations play important roles in cancer progression and therapy [58]. Hence, there have been efforts aimed at mapping the single-cell lncRNA landscape of T cells towards a better understanding of T cell regulation in cancer immunity and immunotherapies [59]. Both cancer and immune cells undergo metabolic reprogramming during tumorigenesis and cancer progression [60,61]. It is reported that lncRNAs can regulate both metabolism and the immune microenvironment through generation and utilization of metabolites [62]. Roles of lncRNAs in cancer glucose metabolism via regulation of various glucose transporters, kinases and metabolic pathways have also been noted [63]. Moreover, multiple studies have suggested key roles for lncRNAs *MALAT1*, *LINC-PINT* and *NEAT1* in cancer [64], and in therapeutic resistance in breast cancer [65]. Recently, a CRISPR-based pipeline using non-small cell lung cancer (NSCLC) suggested that cancer vulnerabilities can be targeted by potent antisense oligonucleotide inhibitors [66]. Studies have also suggested potential roles of lncRNAs as biomarkers in cancer diagnosis such as prostate cancer antigen 3 (PCA3) for prostate cancer and EGFR-AS1 and MIR205HG as predictors of antiepidermal growth-factor receptor drug response [67,68]. Tumor drug resistance, which is a formidable obstacle in successful chemotherapy, can be modulated by lncRNAs in several ways, such as regulation of gene expression, alteration of drug metabolism, drug efflux, drug targets, epithelial–mesenchymal transition, and inhibition of drug-induced apoptosis [69]. These studies have indicated extensive roles of lncRNAs in cancer therapy [70] and there is an ever-growing discourse on the potential for lncRNAs being targeted for precision therapy in human cancer [71]. Concurrently, there has been an emergence of curated repositories that catalogue lncRNA-cancer associations. For instance, Lnc2RNA is a database of experimentally characterized associations between lncRNAs and various human cancers [72]. Another repository, Cancer LncRNA Census (CLC) leverages direct functional or genetic evidence to assign causal lncRNA-cancer roles for 122 GENCODE lncRNAs with established roles in cancer processes [49]. The well-characterized roles of lncRNAs in various cancer types are summarized in Table S1.

## 3. Molecular Functions of NRs and Their Roles in Cancer

### 3.1. Functional Repertoire of NRs

NRs are typically composed of an N-terminal domain (NTD), a DNA binding domain (DBD), a hinge region, a ligand-binding domain (LBD) and a C-terminal domain [73]. A large number of NRs have been discovered as a part of multimember families in different species (for instance, 48, 49, 49 and 47 NRs have been reported in human, mouse, rat and dolphin, respectively), and they are broadly divided into four subgroups [74,75]. Type I receptors such as androgen receptors (ARs) and progesterone receptors (PRs) bind to direct repeats as homodimers upon ligand activation and regulate the expression of target genes involved in cellular physiology and pathophysiology [21,76,77]. Type II NRs such as peroxisome-proliferation-activated receptor (PPAR) and retinoid acid receptor (RAR) form heterodimers with retinoid X receptor (RXR) upon activation and translocate to specific DNA motifs (AGGTCA) termed PPAR response elements (PPRE). PPARs bind to the 5′ end of the PPRE, while RXR binds to the 3′ end [78]. These complexes regulate diverse functions including lipid metabolism, energy homeostasis, gene expression, differentiation, proliferation and apoptosis and inflammatory conditions [79,80,81,82,83,84]. PPAR superfamily comprises three subtypes, namely, PPARα, PPARβ/δ and PPARγ, which are encoded by separate genes and display differential tissue distribution [85]. Apart from fatty acids and their metabolites, PPARs are also activated by several hypolipidemic and antidiabetic drugs via binding with the ligand-binding domain (LBD), which causes changes in their conformation and allows them to regulate the expression of their target genes [83,84,86]. The number of carbon atoms, degree of saturation and configuration of fatty acids play important roles in the activation of PPARs [87]. Type III NRs bind to direct repeat and palindromic sequences as homodimers and can regulate constitutive transactivation and transrepression functions without ligand activation [73]. Type IV NRs bind to DNA at a single consensus half-site preceded by a 5′-A/T-rich flanking sequence as monomers [88].

Coregulators are acknowledged to play critical roles in the facilitating NR-regulated transcriptional control [89]. It has also been established that chromatin accessibility, epigenetic modifications and lncRNAs play important roles in the activation of NRs [90]. Further, NR dimerization can play significant roles in gene regulation through their binding to the promoters and/or enhancers of the target genes [91].

### 3.2. NRs in Cancer

**Figure 1:**
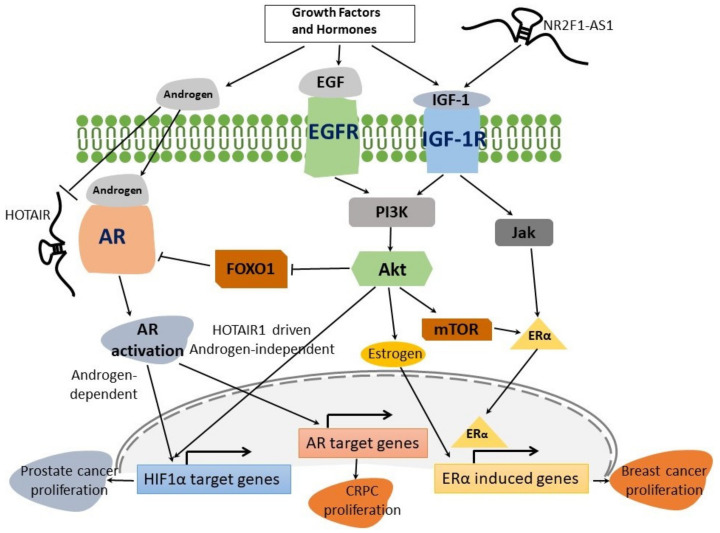
A representative interplay between growth factors, hormones and lncRNAs in cancer proliferation. The binding of the IGF-1 to IGF-1 receptor (IGF-1R) triggers the PI3K/AKT-mTOR and Jak pathways that contribute to increased expression of Erα-induced genes, thereby leading to accelerated breast cancer growth and proliferation. EGFR signaling can also trigger PI3k-AKT signaling, thereby contributing to the activation of Erα-responsive genes. LncRNA *NR2F1-AS1* can also modulate IGF-1R activation by sponging miR-338-3p, a negative regulator of IGF-1. In prostate cancer, IGF-1R signaling activates AR signaling via the IGF-1R-FOXO1 (forkhead box protein O1) signaling axis. AR activation stimulates the expression of HIF1α-induced genes, which contribute to cancer proliferation. HIF1α-responsive genes may also be triggered by EGFR via the PI3K-AKT signaling axis. *HOTAIR* is an androgen-repressed lncRNA that is upregulated in castration-resistant prostate cancer (CRPC); *HOTAIR* associates with AR and drives its androgen-independent activation that subsequently leads to induction of AR-target genes in CRPC proliferation.

NRs such as AR, estrogen receptor (ER), farnesoid X receptor (FXR), glucocorticoid receptor (GR), hepatocyte nuclear factor 4 alpha (HNF4α), liver X receptors (LXR), PPARs and pregnane X receptor (PXR) function as regulators of both inflammation and cancer [92,93,94]. Cross-talks between TLRs and PPARs are known to play important roles in various inflammatory diseases including cancer via modulation of various common signaling pathways such as NF-κB and AP-1 [95]. The roles of steroid receptors AR and ER in both prostate and breast cancers are well established [96]. Indeed, AR and ER coexpression can determine the efficacy of hormone receptor-mediated radiosensitization in breast cancer [97]. Apart from AR and ER, GR-mediated signaling pathways are emerging as key players in breast and prostate cancers [98]. The GR-related signaling can lead to either cancer progression or cancer suppression in different cancer types [99]. It has been shown that GR activation can also trigger anticancer drug tolerance via modulation of the *CDKN1C* gene in lung cancer [100]. In particular, the interplay among hormones, growth factors such as EGF (epidermal growth factor)- and IGF (insulin-like growth factor)-mediated signaling and even lncRNAs, plays an important role in the pathogenesis of many cancer types such as breast and prostate cancer [101,102,103,104,105] (Figure 1). Other widely studied NRs, PPARα, PPARβ/δ and PPARγ, play important roles in both cancer progression and inhibition via modulation of metabolism, inflammation, cell proliferation and apoptosis in various cancer types in a context-dependent manner [106,107,108]. Many studies have suggested potential roles of PPAR modulators in cancer therapeutics [109,110]. NR HNF4α is involved in the regulation of various biological processes, for instance, inflammation, cell proliferation, and drug and lipid metabolism [111]. Also, the tumor-promoting role of HNF4α through induction of cell proliferation, invasion, metastasis and angiogenesis is well studied [112]. The tumor-suppressor role of HNF4α through the regulation of p21-mediated cellular senescence [113] in prostate cancer is also reported. HNF4α is a promising therapeutic target in a variety of cancers via the regulation of various signaling pathways (i.e., Wnt/β-catenin, NF-κB, STAT3, TGFβ) [94]. LXRs are expressed in cancer, stromal and immune cells, and can regulate various signaling pathways involved in lipogenesis, inflammation, cell cycle, and metabolism [114]. Therapeutic roles of LXR ligands via modulation of cholesterol homeostasis in various cancer types such as breast, glioblastoma and melanoma are emerging [115,116,117]. The FXR nuclear receptor is activated by bile acids and plays important roles in the regulation of metabolism [118]. Expression and functional roles of FXR in various types of cancers such as breast, pancreatic, gastric, cervical, colon and hepatocellular carcinoma have been reported [119]. In addition to the regulation of metabolism, FXR regulates proliferation and apoptosis in cancer cells [119,120]. It was demonstrated that the overexpression of FXR can inhibit migration, adhesion and angiogenesis of bladder cancers through proteasome degradation and VEGF reduction [121]. Furthermore, various studies have suggested a therapeutic potential for FXR-regulated targets such as matrix metalloproteinase 7 in colon, and Wnt/β-catenin in colorectal cancer, respectively [122,123]. PXR is an orphan NR that is activated by both endobiotics and xenobiotics, which can be expressed in several cancer types, such as prostate, breast, cervical and ovarian [124]. PXR can regulate various important cellular events such as proliferation, cell cycle, apoptosis, metabolism and chemotherapeutic drug resistance [125]. Roles of PXR polymorphisms in the chemotherapy of cancer patients have also been suggested [126]. A list of NRs with well-characterized roles in various cancer types is summarized in Table S1.

## 4. Cross-Talks between lncRNAs and NRs in Cancer

**Figure 2:**
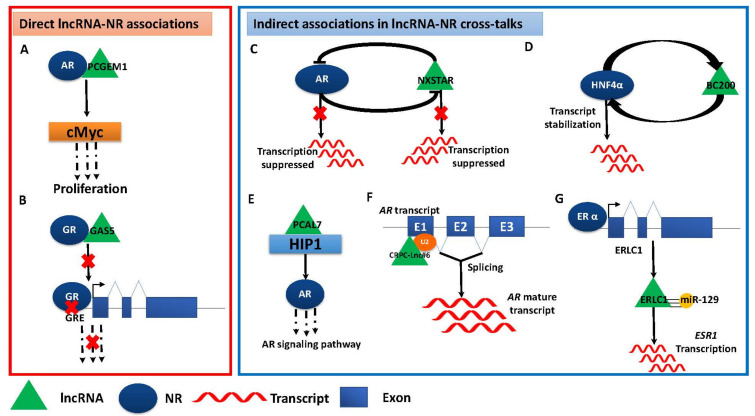
Mechanisms underlying cross-talks between lncRNAs and NRs. lncRNAs and NRs can mutually regulate each other through direct physical associations: (**A**) Transcriptional coactivation—*PCGEM1* functions as a coactivator for AR and cMyc and promotes proliferation. (**B**) Decoy binding—*GAS5* binds to the GR DBD as a GRE decoy and limits its binding to GREs in the target genes. LncRNAs and NRs may also mutually regulate or interact indirectly with each other in different ways. (**C**) Negative feed-forward circuitry—*NXSTAR* and AR mutually negatively impact transcription. (**D**) Feedback regulation—positive feedback loop *BC200* stabilizes *HNF4α* mRNA. (**E**) Activation of signaling—*PCAL7* stabilizes HIP1 and activates AR signaling. (**F**) Regulation of splicing—CRPC-linked lncRNAs associate with splicing factors such U2 to positively regulate *AR* expression. (**G**) Transcriptional activation and miRNA sequestration—Erα transcriptionally activates *ERLC1*; *ERLC1* stabilizes *ESR1* transcript by sequestrating miR-129. Red × means that because of the preceding event, the downstream process is stopped from proceeding further.

Many roles of lncRNAs and NRs in cancer networks are driven by cross-talks between them; these cross-talks determine gene expression, regulate metabolism and signaling pathway outcomes and modulate immune resistance in various types of cancer. The cross-talks between lncRNAs and NRs may happen in many different ways, such as transcriptional regulation, coactivation, chromatin reorganization, interplay between the shared constituents of signaling pathways, etc., and they regulate many different biological processes, including those associated with human disorders such as atherosclerosis, Alzheimer’s disease, various cancers [52] and liver diseases [127] (Figure 2 and Table 1).

LncRNA-NR cross-talks may be viewed as broadly falling into two mechanistic classes. The first category includes instances where lncRNAs and NRs associate with each other via direct physical contacts (Figure 2A,B); it is well established that apart from direct activation by ligands, NRs can also be regulated by direct interactions with lncRNAs and in turn NRs can also regulate lncRNAs via mutual regulatory feedback loops [147]. The second category involves lncRNAs and NRs functionally regulating each other by modulating gene expression, transcript stabilization, regulation of splicing and cross-talks between lncRNA-mediated and NR-mediated signaling pathways, via overlapping cellular factors (Figure 2C–G). Recently, Cantile et al. have discussed at length the reciprocal interactions between endocrine NRs and lncRNAs in different tumor diseases [148]. Below we discuss well-characterized examples of regulatory associations between individual lncRNAs and specific NRs and their functional outcomes on gene expression and other physiological processes in cancer progression and tumorigenesis.

### 4.1. LncRNA Cross-Talks with ARs

AR, which is activated by binding with circulating androgenic hormones is increasingly implicated with essential roles in various cancer types including prostate cancer pathogenesis and therapy [132] and breast cancer [149]. Several AR-signaling-associated lncRNAs such as *PCAL7*, *PRKAG2-AS1* and *HOXC-AS1* can act as diagnostic biomarkers for prostate cancer [132,150]. Additionally, many lncRNAs such as *PCGEM1*, *CTBP1-AS*, *PCAL7*, *HOTAIR*, *CRPC-Lnc#6*, *SOCS2-AS1*, *NXTAR* and *ARNILA* associate with ARs in different cancers and modulate various aspects of tumorigenesis and carcinogenesis via diverse mechanisms (Figure 2 and Table 1). For instance, Hung et al. demonstrated that prostate cancer gene expression marker 1 (*PCGEM1*), which is an AR-regulated, prostate-specific lncRNA strongly elevated in prostate cancer, regulates metabolic programming in tumor cells by functioning as a coactivator for both AR and c-Myc (Figure 2A) [128]. Takayama et al. showed that positive regulation of AR transcription by *CTBP1-AS* played a significant role in prostate cancer progression [130]. Multiple studies have also established important roles for lncRNA-AR cross-talks in castration-resistant prostate cancer (CRPC), a more aggressive form of prostate cancer; these cross-talks are diverse and involve varied underlying mechanisms. For instance, Li et al. showed that *PCAL7*, an AR-induced lncRNA, binds with and stabilizes Huntingtin-interacting protein 1 (HIP1), which in turn activates AR signaling; this positive feedback loop plays an essential role in CRPC progression (Figure 2E) [132]. Takayama et al. characterized a set of AR-regulated lncRNAs that were upregulated in CRPC tissues and directly modulate the spliceosome complex; since the dysregulation of splicing machinery plays a key role in CRPC progression, lncRNA-mediated perturbations in the spliceosome complex may facilitate CRPC progression (Figure 2D) [133]. Misawa et al. demonstrated that androgen-regulated lncRNA, suppressor of cytokine signaling 2-antisense transcript 1 (*SOCS2-AS1*), which is highly expressed in CRPC, inhibited apoptosis and promoted cancer cell survival. *SOCS2-AS1* also modulated AR signaling by interacting with AR, promoting cofactor recruitment and epigenetic modification of AR-target genes in CRPC [134]. Additionally, Ghildiyal et al. showed that lncRNA *NXSTAR* functions as a tumor suppressor gene, which is negatively regulated by AR and which itself negatively regulates AR and AR-V7 expression in an epigenetic fashion (Figure 2F) [135]. It was also demonstrated that impeding AR binding with lncRNA *SLNCR* blocked melanoma invasion, indicating key oncogenic roles of lncRNA-AR complexes and their implied therapeutic potential [147]. Additionally, in a study from Zhang et al., lncRNA *HOTAIR*, by binding to the AR N-terminal domain (NTD), demonstrably stabilized the AR protein by preventing its ubiquitination and subsequent degradation and contributed to CRPC proliferation [131]. 

Among other cancer types, AR is strongly associated with different forms of breast cancer and the roles of lncRNAs in AR signaling pathways in progression of breast cancer are being increasingly recognized [136,149,151]. Huang et al. built a regression analysis-based risk model to highlight a cluster of androgen-receptor signaling-pathway-related long non-coding RNAs (ARSP-related lncRNAs) that were strongly linked with the incidence and progression of breast cancer and were demonstrated to effectively predict the prognoses of breast cancer patients [151]. Yang et al. identified AR negatively induced lncRNA (*ARNILA*), which is transcriptionally repressed by AR and which itself functions as a sponge RNA for miR-204 to facilitate expression of its target gene *Sox4*, thereby promoting cancer progression, invasion and metastasis in triple negative breast cancer (TNBC) [136].

### 4.2. LncRNA Cross-Talks with ERs

ERs are a group of nuclear-localized or membrane-localized TFs that upon activation by steroid estrogens, bind to estrogen response elements (EREs) and modulate gene expression in various cellular processes and diseases including cancer. ERs and lncRNAs can mutually regulate each other to modulate various processes such as antiestrogen resistance in breast cancer [152]. Estrogen-regulated lncRNAs such as *HOTAIR*, *MALAT1*, *MIAT*, *DSCAM-AS1*, *LINC00472*, *LINC01016*, *EGOT*, *LINC*-*ROR* and *LINP1* can modulate ER-dependent transcriptional changes, histone modifications and enhancer–promoter looping interactions [153] (Table 1). Among specific examples, Liang et al. showed that lncRNA DLGAP1 antisense RNA 2 (*DLGAP1-AS2*) was significantly upregulated in ER-positive breast cancers. *DLGAP1-AS2* upregulation facilitated increased ER-signaling via the inhibition of AFF3 degradation, which in turn increased cell viability, inhibited apoptosis and induced resistance to the drug tamoxifen; *DLGAP1-AS2* therefore offered a promising target for anti-breast cancer therapy aimed at overcoming tamoxifen resistance [137]. Miano et al. showed that ER-regulated lncRNA Down syndrome cell adhesion molecule antisense 1 (*DSCAM-AS1*) was highly expressed in Erα+ breast carcinoma and its expression correlated with EMT and invasiveness, suggesting that ER-*DSCAM-AS1* interplay may play an important role in breast cancer [138].

ER may also modulate lncRNA transcription via attaching to genomic binding sites; Fang et al., for instance, highlighted an oncogenic role for estrogen-inducible lncRNA (*ERINA*) in ER-positive breast cancer. *ERINA* was transactivated by ER via an intronic ER-binding region and it promoted tumor proliferation and cell-cycle progression chiefly via binding to and preventing the interaction of TF E2F1 with tumor suppressor retinoblastoma protein (RB1). Since *ERINA* knockdown impeded cancer progression and tumorigenesis, it is a promising candidate as a breast cancer biomarker and target for anti-breast cancer therapy [139].

In contrast, the roles of lncRNAs in directly regulating ER-activity have also been reported. Yuan et al. performed a computational analysis of NGS data for ER-regulated and breast cancer-related lncRNAs and observed that Erα-regulated lncRNA 1 (*ERLC1*), a breast tissue specific lncRNA, was transcriptionally activated by Erα and in turn *ERLC1* stabilized estrogen receptor 1 (*ESR1*) transcript to modulate Erα signaling, therefore emerging as a promising therapeutic target in breast cancer (Figure 2G) [140]. Among other examples, functional roles of lncRNA *TMPO-AS1* in breast cancer progression via the stabilization of ESR1 mRNA expression have been reported [154]. It was also observed that ER-regulated lncRNA *LINC00263* is differentially expressed in male and female patients [155]; these examples have indicated that ER-lncRNA-ESR1 interplay offers a promising avenue for targeted anti-breast cancer therapies.

### 4.3. LncRNA Cross-Talks with GRs

GR is a cytosol-localized factor that upon binding by glucocorticoids, either activates gene expression in the nucleus or prevents the translocation of other TFs to the nucleus, thereby repressing gene expression. As a quintessential example of a direct physical interaction-mediated lncRNA-NR cross-talk (Figure 2B), Kino et al. reported that lncRNA Gas5 can function as a GRE decoy by binding to the GR DNA-binding domain (DBD) and subsequently limiting GR binding to glucocorticoid response elements (GREs) in the target genes; this Gas5-GR interplay was demonstrated to modulate GR activity in HeLa cancer cells [156].

### 4.4. LncRNA Cross-Talks with PPARs

PPARs play important roles in regulating carbohydrate and fatty acid metabolism and are also implicated in many cancer types. LncRNA-PPAR cross-talks have been implicated in the progression of Hepatocellular carcinoma (HCC) (Table 1). Specifically, PPARα directly binds to the lncRNA *Gm15441* promoter to upregulate its expression and attenuate hepatic inflammation by decreasing the NLR family pyrin domain containing 3 (NLRP3) inflammasome activation [157], which is known to play important roles in cancer [158]. Furthermore, the inhibition of PPARα enrichment on the lncRNA-APC promoter enforced its expression and inhibited colorectal carcinoma pathogenesis via the reduction in exosome production [159]. It was recently reported that lncRNA small nucleolar RNA host gene 1 (*SNHG1*) is expressed in several cancer types such as breast, colorectal, pancreatic and neuroblastoma [160,161,162] as a competitive endogenous RNA and can inhibit PPARγ expression to promote bladder cancer pathogenesis [163]. The role of lncRNA *MALAT1* in cancer-associated cachexia through inhibition of adipogenesis via regulation of PPARγ has also been reported [164]. *HULC* (highly upregulated in liver cancer) is an lncRNA specifically overexpressed in HCC; *HULC* upregulates PPARα to activate acyl-CoA synthetase subunit ACSL1, thereby aberrantly modulating lipid metabolism in hepatoma cells and in the development of HCC [141].

### 4.5. LncRNA Cross-Talks with HNF4α

Hepatocyte nuclear factor 4 alpha (HNF4α) is the most abundant DNA-binding protein in liver and binds to DNA as a homodimer to regulate expression of genes in lipid metabolism; aberrant expression of HNF4α is a prominent feature in many diseases including cancer. Various lncRNAs can associate with HNF4α to modulate tumor progression in different cancer types (Table 1). Specifically, lncRNAs, such as *LINC00858* in colon cancer, *LOC100996425* in prostate cancer and LINC00511 in colorectal cancer, were shown to induce tumorigenesis and cancer progression via regulation of HNF4α and associated signaling pathways such as AMPK/mTOR [165,166,167]. Deng et al. showed that lncRNA *SNHG16*, which is involved in the pathogenesis of neuroblastoma, regulated *miR-542-3p*/HNF4 axis via the RAS/RAF/MEK/ERK signaling pathway [168]. Further, Jin et al. demonstrated that lncRNA *HOTAIR* can recruit SNAIL to HNF4α promoter to inhibit its expression and promote epithelial–mesenchymal transition of colorectal cancer [169]. Studies have also indicated that lncRNA-HNF4α cross-talks could be a therapeutic target for anticancer strategies. Chen et al. demonstrated that in invasive mucinous lung adenocarcinoma (IMA), HNF4α transactivated lncRNA *BC200* (brain cytoplasmic 200 lncRNA), which in turn functioned as a scaffold for the fragile X messenger ribonucleoprotein 1 (FMR1), an mRNA-binding protein that stabilizes cancer-related mRNAs and HNF4α mRNA in a positive feedback loop (Figure 2D). Furthermore, mycophenolic acid (MPA), a component of the FDA-approved drug MMF (mycophenolate mofetil), functioned as an HNF4α antagonist to impede the progression of IMA, possibly by disrupting the HNF4α-*BC200*-FMR1 circuit and thereby providing a promising avenue for anti-IMA therapy [143].

### 4.6. LncRNA Cross-Talks with RAR

RARs form heterodimers with RXRs upon retinoid binding and bind to retinoid acid response elements (RARE) in the promoters of their target genes [170]. Some lncRNAs are known to associate with RAR signaling in cancer. Among the earliest examples, Zhao et al., using NB4, a promyelotic cell line, showed that the expression of HOXA cluster antisense RNA 2 (*HOXA-AS2*) was induced by RAR signaling in acute promyelocytic leukemia (APL), which in turn suppresses pro-apoptotic pathways [144]. Likewise, Haji et al. demonstrated that lncRNA *H19* expression was upregulated in APL by RAR signaling, which in turn disrupted telomerase activity by interfering with the interaction between telomerase reverse transcriptase (hTERT) enzyme and telomerase RNA component (hTR), thereby contributing to tumor suppression [145]. Therefore, H19 offers an attractive target for therapeutic intervention in AML and related cancer types. Further examples of RAR/RXR-lncRNA cross-talks are summarized in Table 1. For a more detailed perspective of lncRNA-RAR/RXR cross-talks in cancer, readers are directed to refer to the review by Cantile et al. [148].

### 4.7. LncRNA Cross-Talks with LXR

Liver X receptor (LXR) belongs to a family of cholesterol ligand-activated TFs that are significant modulators of fatty acid and glucose metabolism. Wan et al. reported that an LXR agonist, LXR-623-induced lncRNA *LINC01125*, can suppress proliferation of breast cancer cells through the PTEN/AKT/p53 signaling pathway. This observation suggested that targeting NRs using receptor agonists such as LXR-623 may be a useful strategy for antitumor therapies [171].

### 4.8. LncRNA Cross-Talks as Therapeutic Targets

Therapeutic modulation of lncRNA-NR contacts to control the downstream processes and impede or even reverse cancer proliferation is an attractive, yet challenging task. There are multiple studies and applications towards targeting lncRNAs and NRs in diseases; in one such study, He at al. demonstrated the therapeutic potential for the antiangiogenic drug Sunitinib, which is widely used for renal cell carcinoma (RCC) therapy. The study showed that Sumatinib enhanced the expression of *ECVSR* lncRNA, thereby stabilizing ERβ mRNA. This stabilizing effect resulted in an increased *ECVSR*-ERβ-Hif2α signaling, leading to increased cancer stem cell (CSC) phenotype and vascular mimicry formation of RCC cells [172].

## 5. Multiomics Approach to Characterizing the Regulatory Networks Underlying lncRNA-NR Cross-Talks

**Figure 3:**
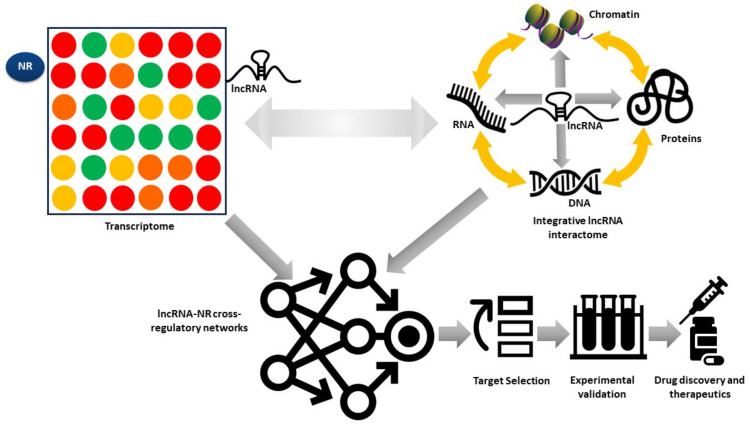
High-throughput omics technologies can provide heterogeneous lncRNA and biomolecular interaction data; these data can be assembled into an lncRNA-centric interactome that in conjunction with gene expression data may then be used to generate lncRNA-NR gene regulatory networks (GRNs), to hypothesize the mechanisms underlying lncRNA-NR interactions and their phenotypic outcomes. These outcomes can then be leveraged to rank and prioritize biologically and therapeutically relevant candidates for biomedical and drug-target discovery.

While many lncRNAs-NR cross-talks in cancer have been individually characterized, a clear understanding of the general mechanisms underlying these associations remains elusive. LncRNAs are implicated in the regulation of gene expression typically via biomolecular interactions [173], and lncRNA-NR cross-talks, like all cellular networks, will often encompass different organizational levels involving interactions between RNAs, DNA/chromatin, proteins and small molecule metabolites, among others. Therefore, properly characterizing lncRNA-NR cross-talks is a non-trivial task. To actively advance our understanding of the lncRNA-NR cross-regulatory networks in cancer, these associations should preferably be visualized through multiple lenses. To achieve this, it is necessary to employ a holistic multiomics approach combining genome-wide RNA-chromatin interactions, chromatin–chromatin (DNA-DNA) interactions, protein–protein interactions (PPIs), RNA–protein interactions (RPIs), RNA-RNA interactions and small molecule (chemical) interactions with global gene expression (transcriptome) data to assemble lncRNA-NR gene regulatory networks (GRNs) to characterize lncRNA-NR cross-talks in different cellular contexts, pinpoint key modulators of these processes and prioritize candidates for experimental validation, biomarker discovery and therapeutic intervention in diseases such as cancer (Figure 3).

### 5.1. High-Throughput Omics Methodologies

Different omics platforms provide distinct high-throughput data on different organizational levels of a cell or an organism. Therefore, an integrative multiomics approach that leverages multiple omics data-types together with bioinformatics tools for data analysis and pattern discovery is well suited to characterize different layers of lncRNA-NR cross-regulatory networks.

### 5.2. Transcriptome

The entirety of the RNA population within a cell, tissue or a living system is referred to as the transcriptome and the study of the transcriptome is known as transcriptomics. The two most widely used techniques to profile the transcriptome are microarrays and RNA-seq [174]. Both lncRNAs and TFs such as NRs display cell-specific expression; hence, global transcriptome profiling can help identify sets of coexpressed lncRNAs and NR-coding genes in specific cancer types (Figure 3). However, it is also well established that lncRNAs, in general, have more restricted expression than protein-coding messenger RNAs (mRNAs); this is in part attributed to high cellular specificity of lncRNA expression and disproportionately high expression of lncRNAs in cellular subpopulations [1]. Therefore, global transcriptome profiling coupled with single-cell RNA-seq analysis is well suited to profile lncRNA and TF (including NRs) coexpression in specific cancer types. One such initiative is the RNA Atlas, which combines multiple RNA sequencing technologies together with lncRNA-target prediction to expand the catalog of heterogeneous transcripts in the human transcriptome and analyze correlations with lncRNAs and TFs such as NRs [175]. 

### 5.3. Biomolecular Interactions Underlying lncRNA-NR Cross-Talks

Various high-throughput experiments can offer detailed insights into the different types of biomolecular associations that typically underlie lncRNA-NR cross-talks. Technologies such as cross-linking and immunoprecipitation (IP) followed by sequencing (CLIP-seq) and associated methods can potentially capture direct binding between RNAs such as lncRNAs and proteins such as NRs [176]. Gene regulatory interactions between lncRNAs and NRs can be profiled on a genome-wide scale by using technologies that capture protein–DNA interactions [177] and those that profile RNA–chromatin interactions [27]; these include one-to-many methods such as CHIRP-seq [178] and CHART-seq [179] and many-to-many methods such as RADICL-seq [180] and GRID-seq [181], among others. PPIs between NRs and other cellular proteins can be mapped using different experimental techniques such as the yeast two-hybrid system (Y2H) [182], affinity purification–mass spectrometry (AP-MS) for one-to-many PPI mapping [183] and cofractionation and mass spectrometry [184] and PROPER-seq, for the mapping of many-to-many PPIs [185]. Chromatin–chromatin interactions involving lncRNA and NR loci can be mapped using chromosome conformation capture techniques such as Hi-C [186], while lncRNA-NR mRNA interactions can be characterized with methods such as RIC-seq [187].

### 5.4. Computational Tools

Computational and statistical methods to assess huge volumes of genomics and transcriptomics data have been pivotal to new discoveries in RNA biology. For instance, computational methods such as catRAPID [188] can be used to rapidly predict RNA-protein (NR) interactions on a global scale, which can then be prioritized for experimental validation. Likewise, in silico methods for PPI prediction [189,190] can complement and qualitatively assess PPIs likely to play important roles in lncRNA-NR cross-talks. However, individual analytical methods, though useful in their own right, will provide only specific insights into the many layers that make up the lncRNA-NR cross-regulatory networks. A multiomics data analysis approach, which seeks to combine different omics data types and different tools for their analysis into a unified analytical framework, is better suited to understand the depth of lncRNA-NR cross-talks. These methods for data classification and categorization can take many forms and differ in scope and content but can broadly be viewed as falling into two categories—unsupervised (such as clustering [191] and matrix factorization [192]) and supervised (such as network modeling [193] and most machine-learning algorithms [194]). For a detailed overview of integrative omics methods for building lncRNA gene regulatory networks and knowledge discovery, readers are directed to reviews by Tripathi et al. [195] and Zhao et al. [2].

## 6. Conclusions and Perspectives

LncRNAs can regulate NR signaling either individually or in conjunction with other coregulators in a context-dependent manner and in feedback loops. Multiple studies have increasingly highlighted the cross-talks between lncRNAs and NRs in the regulation of chromatin modifications and cellular processes involved in tumor progression, resistance and metastasis. Emerging studies have also highlighted the roles of lncRNA-NR cross-talks in metabolic reprogramming of cells, which can lead to cancer progression. Dysregulation of NR-mediated signaling is observed in a variety of hormone-dependent cancers. Studies have also suggested roles of NR-associated lncRNAs in cancer as prognostic biomarkers and in the development of novel treatment options. Since most of the genome is composed of lncRNAs, exploring lncRNAs and NR-interactions can provide a wider range of prognostic and therapeutic opportunities, especially in the treatment of hormone-dependent cancers such as breast and prostate cancers. A deeper understanding of lncRNA-NR cross-talks can best be obtained from a combined approach that integrates different data types in a multiomics framework. Consequently, computational tools that can facilitate integrative analysis of multidimensional omics data to infer representative lncRNA-NR regulatory networks will help gain more knowledge about these associations and help prioritize targets for experimental and clinical validation.

## Figures and Tables

**Table 1:** Selected NR and lncRNA cross-talks and their functional roles in cancers.

NRs	lncRNAs	Cancer Type	Mechanisms	Impact on Cancer Pathogenesis	Refs.
AR	*PCGEM1*	Prostate	Coactivator for AR and c-Myc, tumor metabolism and carcinogenesis	Promotes cancer progression.	[128,129]
	*CTBP1-AS*	Prostate	Promotes AR transcription and cell cycle.	Promotes cancer progression.	[130]
	*HOTAIR*	CRPC	Binds to N-terminal domain of AR, preventing its ubiquitination and degradation.	Enhanced cell migration and proliferation.	[131]
	*PCAL-7*	CRPC	Interacts with and stabilizes HIP1 protein to activate AR signaling.	Promotes cell growth and invasion and cancer proliferation.	[132]
	*CRPC-Lnc#6*	CRPC	Modulates AR splicing.	Abnormalities in target gene expression.	[133]
	*SOCS2-AS1*	CRPC	Apoptosis-inhibition.	Enhanced cell growth and migration.	[134]
	*NXTAR*	CRPC	Inhibits AR/AR-V7; AR negatively regulates NXTAR in a negative feed-forward *NXTAR*-AR circuitry.	Loss of *NXTAR* leads to enhanced tumorigenesis.	[135]
	*ARNILA*	Triple Negative Breast Cancer (TNBC)	AR suppresses *ARNILA* transcription by promoter binding	Sequesters miR-204 leading to upregulation of Sox4, promotes EMT, invasion and metastasis.	[136]
ERα	*DLGAP1-AS2*	Breast	Binds to and inhibits AFF3 degradation, promoting ER signaling.	Confers tamoxifen resistance.	[137]
	*DSCAM-AS1*		Erα positively regulates *DSCAM-AS1* expression.	Correlates inversely with EMT markers; cancer progression.	[138]
	*ERINA*		ER transactivates *ERINA* via intronic binding site; *ERINA* binds to E2F1 and prevents interaction with RB1.	Promotes cell-cycle progression and tumor proliferation; correlated with poor patient survival.	[139]
	*ERLC1*		Erα transcriptionally activates *ERLC1*; *ERLC1* stabilizes *ESR1* transcript by sequestrating miR-129.	Promotes resistance to antiestrogen therapies in breast cancer.	[140]
PPARα	*HULC*	Hepatocellular carcinoma	Deregulates lipid metabolism via *miR-9*, PPARA, and ACSL1 pathway.	Malignant development.	[141]
PPARγ	*Ftx*		Aerobic glycolysis	Tumor progression.	[142]
HNF4α	*BC200*	Invasive mucinous lung adenocarcinoma (IMA)	HNF4α-*BC200*-FMR1 positive feedback loop stabilizes cancer-related and HNF4α mRNAs.	Tumor growth and metastasis.	[143]
RAR/RXR	*HOXA-AS2*	Acute promyelocytic leukemia (APL)	RAR signaling upregulates *HOXA-AS2*, which in turn reduces caspase expression.	Tumor growth and metastasis.	[144]
	*H19*	APL	RAR signaling upregulates H19, which perturbs telomerase activity.	Tumor growth and metastasis.	[145]
	*RAET1K*	Lung	*RAET1K* counteracts RA activity by sponging *miR-135a-5p*, thereby enhancing Cyclin E1 expression.	Cell-cycle progression in tumorigenesis.	[146]

## Supplementary Materials

The following supporting information can be downloaded at: https://www.mdpi.com/article/10.3390/cancers16162920/s1, Table S1: Selected lncRNAs and NRs associated with cancers.
