# Supplementary material for: Long Non-Coding RNAs, Nuclear Receptors and Their Cross-Talks in Cancer—Implications and Perspectives
Source: Cancers (Basel). 2024 Aug 22;16(16):2920. doi: 10.3390/cancers16162920 (PMC11352509; doi:10.3390/cancers16162920)
Supplement: Supplementary file 1 [file cancers-16-02920-s001.zip › cancers-3068531-supplementary.pdf]

Table S1: Selected lncRNAs and NRs associated with cancers.

LncRNAs	Cancer type	Mechanisms	Impact on cancer pathogenesis	References
MALAT1	Breast	Activation of PI3K/AKT pathway	Induces EMT-like phenotype and cell invasiveness.	[1]
		Downregulation of TALAM1	Increases cell proliferation, migration and tube formation.	[2]
		Downregulation of mi-145	Cell aggressiveness and malignancy	[3]
	Cervical	Activation of PI3K/AKT pathway	Induces EMT-like phenotype.	[4]
		Activation of AKT/mTOR pathway	Enhances cell migration and invasion, EMT.	
		Suppression of miR-124 expression	Cervical carcinoma proliferation	[5]
	Colorectal	Modulates Wnt/β-catenin, YAP, SOX9, RUNX2, Snail, EGF, PI3K/AKT/mTOR, and VEGF pathways; modify miRNAs associated with drug sensitivity.	Induces cancer progression and tumorigenesis.	[6]
	Endometrial	Activation of Wnt/β-catenin pathway	Induces cell migration and invasion.	[7]
	NSCLC	Regulates GINS1 expression via FOXP3.	Promotes cancer proliferation.	[8]
		Functions as miRNA sponge	Promotes proliferation, progression, invasion and tumor formation.	[9] (and references within)
		Activation of PI3K/AKT pathway	Promotes tumor proliferation and metastasis.	[4]
				[10]
	Ovarian	Activation of PI3K/AKT, Wnt/β-catenin pathways.	Induces EMT-like phenotype.	[11]
		Inhibits activation of JAK/STAT pathway by targeting miR-503-5p	Promotes proliferation, apoptosis inhibition.	[12]
		Regulation of RBOX-2 mediated alternative splicing.	Facilitates pro-metastatic phenotype, cancer progression.	
				[13]
	Prostate	Downregulation of miR-1	Promotes proliferation and migration, apoptosis inhibition.	[14]
		Regulation of miR-145-5p-SMAD3/TGFBR2 interactions	Promotes EMT, cancer progression	
DSCAM-AS1	Multiple cancer types	Interactions with diverse miRNAs and proteins; modulates NOTCH, Wnt/β-catenin pathways.	Cancer progression, clinical deterioration, therapeutic resistance.	[15]
FTX	Hepatocellular carcinoma	Aerobic glycolysis.	Proliferation, invasion and migration.	[16]
HNF4A-AS1	Neuroblastoma	Binds to hnRNPU protein and activates genes associated with tumor progression.	Promotes aerobic glycolysis tumor progression and aggressiveness.	[17]
HOTAIR1	Multiple cancer types	Lipid metabolism.	Proliferation, invasion and migration.	[18]
NEAT1	Prostate	Upregulated by Erα, in turn upregulates prostate cancer genes	Promotes cancer progression and tumorigenesis.	[19]
**NRs**				
AR	Prostate	Interacts with FOXA1 as a co-factor; directly interacts with PTEN, inversely correlates with *PTEN* expression.	Drives cell cycle progression from G2 to M stage and tumor growth and progression.	[20]
	Breast	Positively correlates with *PTEN* expression.	Lowered *PTEN* expression correlates with tumor progression.	[21]
ERα	Multiple cancer types	Modulates PI3K/AKT/mTOR/ NF-κB, MAPK/ERK signaling pathways	Carcinogenesis, tumor invasion, metastasis.	[22]
HNF4α	Multiple cancer types	Modulation of Wnt/β-catenin, NF-κB, STAT3 3, and TGFβ signaling.	Occurrence, progression, proliferation.	[23,24]
PPARα PPARγ	Hepatocellular carcinoma	Modulation of cell cycle, induction of apoptosis,.	Deficiency leads to increased carcinogenesis, cell proliferation.	[25,26]

References

1. Arun, G.; Spector, D.L. MALAT1 long non-coding RNA and breast cancer. *RNA Biology* **2019**, *16*, 860-863, doi:10.1080/15476286.2019.1592072.
2. Qiao, F.-H.; Tu, M.; Liu, H.-Y. Role of MALAT1 in gynecological cancers: Pathologic and therapeutic aspects (Review). *Oncol Lett* **2021**, *21*, 333, doi:10.3892/ol.2021.12594.
3. Malakoti, F.; Targhazeh, N.; Karimzadeh, H.; Mohammadi, E.; Asadi, M.; Asemi, Z.; Alemi, F. Multiple function of lncRNA MALAT1 in cancer occurrence and progression. *Chemical Biology & Drug Design* **2023**, *101*, 1113-1137, https://doi.org/10.1111/cbdd.14006.
4. Zhao, M.; Wang, S.; Li, Q.; Ji, Q.; Guo, P.; Liu, X. MALAT1: A long non-coding RNA highly associated with human cancers.
5. Liang, T.; Wang, Y.; Jiao, Y.; Cong, S.; Jiang, X.; Dong, L.; Zhang, G.; Xiao, D. LncRNA MALAT1 Accelerates Cervical Carcinoma Proliferation by Suppressing miR-124 Expression in Cervical Tumor Cells. *J Oncol* **2021**, *2021*, 8836078, doi:10.1155/2021/8836078.
6. Xu, W.-W.; Jin, J.; Wu, X.-y.; Ren, Q.-L.; Farzaneh, M. MALAT1-related signaling pathways in colorectal cancer. *Cancer Cell International* **2022**, *22*, 126, doi:10.1186/s12935-022-02540-y.
7. Zhao, Y.; Yang, Y.; Trovik, J.; Sun, K.; Zhou, L.; Jiang, P.; Lau, T.S.; Hoivik, E.A.; Salvesen, H.B.; Sun, H.; et al. A novel wnt regulatory axis in endometrioid endometrial cancer. *Cancer Res* **2014**, *74*, 5103-5117, doi:10.1158/0008-5472.can-14-0427.
8. Li, M.; Shi, M.; Hu, C.; Chen, B.; Li, S. MALAT1 modulated FOXP3 ubiquitination then affected GINS1 transcription and drived NSCLC proliferation. *Oncogene* **2021**, *40*, 3870-3884, doi:10.1038/s41388-021-01816-3.
9. Zhou, Q.; Liu, L.; Zhou, J.; Chen, Y.; Xie, D.; Yao, Y.; Cui, D. Novel Insights Into MALAT1 Function as a MicroRNA Sponge in NSCLC. *Front Oncol* **2021**, *11*, 758653, doi:10.3389/fonc.2021.758653.
10. Jin, Y.; Feng, S.J.; Qiu, S.; Shao, N.; Zheng, J.H. LncRNA MALAT1 promotes proliferation and metastasis in epithelial ovarian cancer via the PI3K-AKT pathway. *Eur Rev Med Pharmacol Sci* **2017**, *21*, 3176-3184.
11. Sun, Q.; Li, Q.; Xie, F. LncRNA-MALAT1 regulates proliferation and apoptosis of ovarian cancer cells by targeting miR-503-5p. *Onco Targets Ther* **2019**, *12*, 6297-6307, doi:10.2147/ott.s214689.
12. Gordon, M.A.; Babbs, B.; Cochrane, D.R.; Bitler, B.G.; Richer, J.K. The long non-coding RNA MALAT1 promotes ovarian cancer progression by regulating RBFOX2-mediated alternative splicing. *Mol Carcinog* **2019**, *58*, 196-205, doi:10.1002/mc.22919.
13. Chang, J.; Xu, W.; Du, X.; Hou, J. MALAT1 silencing suppresses prostate cancer progression by upregulating miR-1 and downregulating KRAS. *Onco Targets Ther* **2018**, *11*, 3461-3473, doi:10.2147/ott.s164131.
14. Zhang, D.; Fang, C.; Li, H.; Lu, C.; Huang, J.; Pan, J.; Yang, Z.; Liang, E.; Liu, Z.; Zhou, X.; et al. Long ncRNA MALAT1 promotes cell proliferation, migration, and invasion in prostate cancer via sponging miR-145. *Transl Androl Urol* **2021**, *10*, 2307-2319, doi:10.21037/tau-20-1526.
15. Ghafouri-Fard, S.; Khoshbakht, T.; Taheri, M.; Ebrahimzadeh, K. A Review on the Carcinogenic Roles of DSCAM-AS1.
16. Li, X.; Zhao, Q.; Qi, J.; Wang, W.; Zhang, D.; Li, Z.; Qin, C. lncRNA Ftx promotes aerobic glycolysis and tumor progression through the PPARγ pathway in hepatocellular carcinoma.
17. Song, H.; Li, D.; Wang, X.; Fang, E.; Yang, F.; Hu, A.; Wang, J.; Guo, Y.; Liu, Y.; Li, H.; et al. HNF4A-AS1/hnRNPU/CTCF axis as a therapeutic target for aerobic glycolysis and neuroblastoma progression. *Journal of Hematology & Oncology* **2020**, *13*, 24, doi:10.1186/s13045-020-00857-7.
18. Tang, Q.; Hann, Swei S. HOTAIR: An Oncogenic Long Non-Coding RNA in Human Cancer. *Cellular Physiology and Biochemistry* **2018**, *47*, 893-913, doi:10.1159/000490131.
19. Chakravarty, D.; Sboner, A.; Nair, S.S.; Giannopoulou, E.; Li, R.; Hennig, S.; Mosquera, J.M.; Pauwels, J.; Park, K.; Kossai, M.; et al. The oestrogen receptor alpha-regulated lncRNA NEAT1 is a critical modulator of prostate cancer. *Nature Communications* **2014**, *5*, 5383, doi:10.1038/ncomms6383.
20. Michmerhuizen, A.R.; Spratt, D.E.; Pierce, L.J.; Speers, C.W. ARe we there yet? Understanding androgen receptor signaling in breast cancer. *npj Breast Cancer* **2020**, *6*, 47, doi:10.1038/s41523-020-00190-9.
21. Vidula, N.; Yau, C.; Wolf, D.; Rugo, H.S. Androgen receptor gene expression in primary breast cancer. *npj Breast Cancer* **2019**, *5*, 47, doi:10.1038/s41523-019-0142-6.
22. Liu, Y.; Ma, H.; Yao, J. ERα, A Key Target for Cancer Therapy: A Review.

23. Lv, D.-D.; Zhou, L.-Y.; Tang, H. Hepatocyte nuclear factor 4α and cancer-related cell signaling pathways: a promising insight into cancer treatment. *Experimental & Molecular Medicine* **2021**, *53*, 8-18, doi:10.1038/s12276-020-00551-1.
24. Sang, L.; Wang, X.; Bai, W.; Shen, J.; Zeng, Y.; Sun, J. The role of hepatocyte nuclear factor 4α (HNF4α) in tumorigenesis.
25. Katoch, S.; Sharma, V.; Patial, V. Peroxisome proliferator-activated receptor gamma as a therapeutic target for hepatocellular carcinoma: Experimental and clinical scenarios.
26. Silva-Gomez, J.A.; Galicia-Moreno, M.; Sandoval-Rodriguez, A.; Miranda-Roblero, H.O.; Lucano-Landeros, S.; Santos, A.; Monroy-Ramirez, H.C.; Armendariz-Borunda, J. Hepatocarcinogenesis Prevention by Pirfenidone Is PPARγ Mediated and Involves Modification of Nuclear NF-kB p65/p50 Ratio. *International Journal of Molecular Sciences* **2021**, *22*, doi:10.3390/ijms222111360.
